# Microparticle-Based Detection of Viruses

**DOI:** 10.3390/bios13080820

**Published:** 2023-08-15

**Authors:** Bradley Khanthaphixay, Lillian Wu, Jeong-Yeol Yoon

**Affiliations:** Department of Biomedical Engineering, The University of Arizona, Tucson, AZ 75721, USA; bradkhant@arizona.edu (B.K.); lillian1117@arizona.edu (L.W.)

**Keywords:** microspheres, latex beads, SARS-CoV-2, Ebola virus, Zika virus, H1N1, hepatitis virus

## Abstract

Surveillance of viral pathogens in both point-of-care and clinical settings is imperative to preventing the widespread propagation of disease—undetected viral outbreaks can pose dire health risks on a large scale. Thus, portable, accessible, and reliable biosensors are necessary for proactive measures. Polymeric microparticles have recently gained popularity for their size, surface area, and versatility, which make them ideal biosensing tools. This review cataloged recent investigations on polymeric microparticle-based detection platforms across eight virus families. These microparticles were used as labels for detection (often with fluorescent microparticles) and for capturing viruses for isolation or purification (often with magnetic microparticles). We also categorized all methods by the characteristics, materials, conjugated receptors, and size of microparticles. Current approaches were compared, addressing strengths and weaknesses in the context of virus detection. In-depth analyses were conducted for each virus family, categorizing whether the polymeric microparticles were used as labels, for capturing, or both. We also summarized the types of receptors conjugated to polymeric microparticles for each virus family.

## 1. Introduction

**Figure 1:**
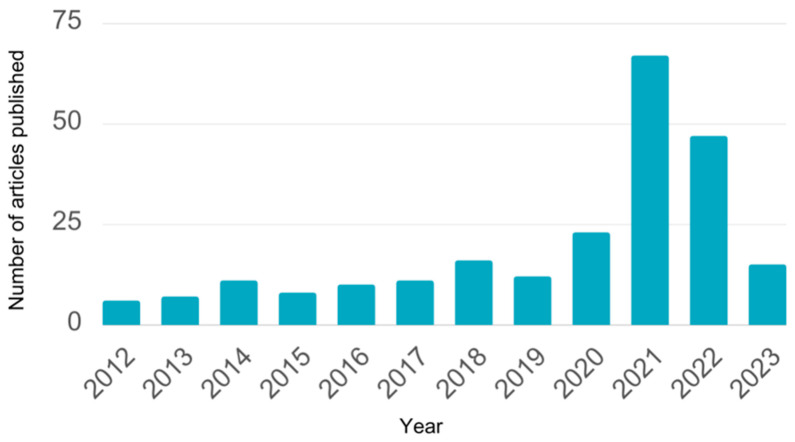
The number of search results for the prompt *microparticle* + *virus* + *detection* on Scopus. Categorized by year and filtered by abstract, title, and keywords.

Viruses significantly burden public health, and viral outbreaks have increased in recent years [1]. Without access to rapid and portable diagnostic tools, virus detection from human specimens and environmental samples becomes increasingly complex, which can delay counteractive measures and containment of the virus’ spread. An untimely response can lead to a cascade of social and financial ramifications, and in the context of a globally connected world, these effects can have widespread implications [2]. Microparticles have recently garnered increased attention due to their suitability for biosensing applications. They can perform various functions, distinguished by their versatility, affordability, and simplicity in many applications [3,4,5]. Most microparticles for biosensing applications are polymers, and we will use the terms polymeric microparticles and microparticles interchangeably in this review. Although the number of research articles published for microparticle-based virus detection platforms has increased significantly over the past five years, coinciding with the onset of the SARS-CoV-2 pandemic (Figure 1), a comprehensive literature review on the uses of microparticles for virus detection has not yet been conducted, especially incorporating the recent updates during the pandemic. In this review, we cataloged the various methods by which microparticles could be applied to virus sensors and compared their effectiveness to current approaches. Additionally, we cataloged typical microparticle-based detection formats by virus families.

Viruses have typically been detected with amplification-based methods, including polymerase chain reaction (PCR) and reverse transcription-PCR (RT-PCR), which are reliable methods for obtaining highly-specific results [6]. The flexible nature of primer design for PCR and its variants allows it to be applied to nearly any viral target, conditional on the currency of the primer [7]. PCR’s typical lack of portability and requirement for large batches makes it challenging to use as a rapid diagnostic tool, as observed during the SARS-CoV-2 outbreak [8]. Moreover, the need for trained personnel, relatively long turnaround time, and imperfect sensitivity can sometimes become additional obstacles [9]. Alternative methods of detecting viral DNA and RNA, such as loop-mediated isothermal amplification (LAMP) and reverse transcription-LAMP (RT-LAMP), can remedy the common pitfalls associated with PCR while maintaining its high specificity. LAMP does not require a typical thermal cycler (required for PCR), and it may reduce the burden of instrumentation. In certain situations, purification steps common to amplification-based methods can be circumvented [10,11]. Significant amplification of target sequences can occur as early as <45 min, and real-time analysis of results can be performed simply by assessing the reaction mixture’s change in turbidity [12]. Although LAMP possesses many advantages that make it ideal for point-of-care (POC) applications, it is heavily constrained by the need for a properly designed primer—even more so than PCR [13].

Non-amplification-based methods present alternative solutions to monitoring viral outbreaks. These include serological tests like hemagglutination-inhibition assays (HIA), immunofluorescence assays, or chemiluminescent immunoassays (CIA). Serological tests typically employ a functionalized system to detect the presence of viral antigens or antibodies for a viral target. They are much simpler, low-cost, and provide much shorter assay time (<15 min) than amplification-based methods. However, less-than-perfect specificity caused by antibody cross-reactivity increases the risk of false-positive results [14]. Therefore, it became a norm to first test with serological immunoassays (rapid antigen tests) and to further confirm the result with amplification-based methods (such as RT-PCR or RT-LAMP) during the SARS-CoV-2 pandemic.

Nevertheless, particle-based modifications to serological tests have been proposed, with results demonstrating high specificity and sensitivity [15,16]. Specifically, metal nanoparticles have emerged as a popular means of improving the selectivity and sensitivity of many non-amplification-based methods [17]. Gold nanoparticles (AuNPs) have most commonly been utilized for numerous serological tests due to their unique properties suitable for virus detection [14]. They can easily be conjugated to antigens, antibodies, proteins, and other biomolecules. When applied in a colorimetric assay, AuNPs can directly label target analytes with a distinct pink color visible to the naked eye, allowing for results to be imaged and analyzed with portable methods [18,19]. Colorimetric detection platforms are often used because of their simplicity. However, higher limits of detection (LODs) are typical limitations compared to other imaging approaches, especially under ambient lighting conditions [20]. Alternatively, quantum dots (QDs; semiconductor nanoparticles) have provided high sensitivity and very low LOD due to their intrinsic photoluminescence or fluorescence [21,22]. 

Microparticles have also been used for numerous serological tests in the past, many of which are polymers, e.g., polymeric microspheres, latex beads, etc. Microparticles present unique ways to modify amplification-based and non-amplification-based viral detection methods. Microparticles can easily be removed from the solution through centrifugation or applying a magnetic field; they can also aid in simplifying and shortening procedures [23]. In addition, microparticles’ greater surface area (compared to conventional surfaces like multiwell plates and lateral flow strips) can be exploited to increase reaction times and target interaction events [24]. It is also possible to incorporate fluorescence into microparticles, allowing for the direct labeling of captured analytes [25].

## 2. Definition and Advantages of Microparticles

Microparticles are spherical particles between 1 and 1000 µm (sometimes 0.1 and 1000 µm) in diameter [26]. These can be hollow or solid and comprise various materials like glass, polymer, ceramics, or metal [27]. They are also called *microspheres* or *microbeads*. Fabrication methods for microparticles vary depending on the size and material of the particle. Many microparticles for virus detection are polymeric microparticles, often called *latex particles* or *latex beads*. Most polymeric microparticles are polystyrene (PS), or co-polymers of PS and other polymers, typically synthesized via emulsion polymerization. Many PS-based microparticles are now commercially available, from companies including MagSphere, Inc. and Bangs Laboratories, Inc. Their sizes range from 0.1 μm to 10 μm, with surface modified types including carboxylated, aminated, sulfonated, etc. (typically by co-polymerization). These commercial PS-based microparticles can also incorporate fluorescent dyes or magnetic properties.

More sophisticated microparticle fabrication technologies have recently been introduced towards more uniform size and improved surface properties. For example, microparticles can be synthesized within a microfluidic device, where emulsion droplets are converted to solid particles through a chemical or photo-polymerization technique [28]. Template-based strategies use a mold to form particles into predefined shapes, but such methods are often limited by how particles can be removed from molds [29,30]. Alternative fabrication techniques have been investigated to produce uniformly and precisely formed particles, including soft lithography, in-fiber fabrication, and electrospraying [31,32,33].

Due to their size properties, microparticles are ideal for centrifugal separation techniques and can be imaged via conventional microscopy, enhancing the simplicity and accessibility of procedures involving microparticles. Lastly, microparticles have been engineered for drug delivery applications, which this review will not cover as it is primarily concerned with virus detection. 

Although similar to microparticles, nanoparticles are primarily distinguished from microparticles in size and electrochemical properties. Nanoparticles are often defined with a diameter of less than 100 nm, and they can be made from the same materials which constitute microparticles, most notably metals and polymers [34]. Due to the nanoscale level of nanoparticles, they are often imaged using high-resolution techniques, such as electron microscopy, atomic force microscopy, etc. Additionally, nanoparticles can be fabricated with diameters equal to or shorter than a single wavelength of light, allowing them to exhibit unique optical and electrochemical properties. These properties have been applied to uses in surface plasmon resonance (SPR) or surface-enhanced Raman spectroscopy (SERS) [35]. Nanoparticles emerged with the advent of modern nanotechnology in the 1980s and have since been used across multiple fields, such as electronic manufacturing, drug delivery, and biochemical sensing [35]. While particles bigger than 100 nm and smaller than 1 μm still belong to the microparticle category, their assignment to microparticles is somewhat ambiguous. Therefore, a third category of *submicron* particles is sometimes used for these particles (100 nm–1 μm). In this review, however, we will consider submicron particles as a part of microparticles.

Both microparticles and nanoparticles have been employed for viral sensing applications. This review, however, will limit the discussion to using microparticles for virus detection.

## 3. How Microparticles Are Used for Virus Detection

Microparticles have previously been used for serological tests, targeting either virus antigens or antibodies generated from immune responses. Typically, antibodies are conjugated to microparticles to detect virus antigens in blood sera, and antigens are conjugated to microparticles to detect neutralizing antibodies in blood sera. The same methods can be duplicated for saliva tests, which have been popularly used during the SARS-CoV-2 pandemic. Such methods can also be used for screening virus antigens in environmental samples, such as aerosols, field water samples, and food samples. However, the number of such virus antigens in environmental samples tends to be very low, requiring very low LODs, which are often difficult to achieve in traditional immunoassays [36].

**Figure 2:**
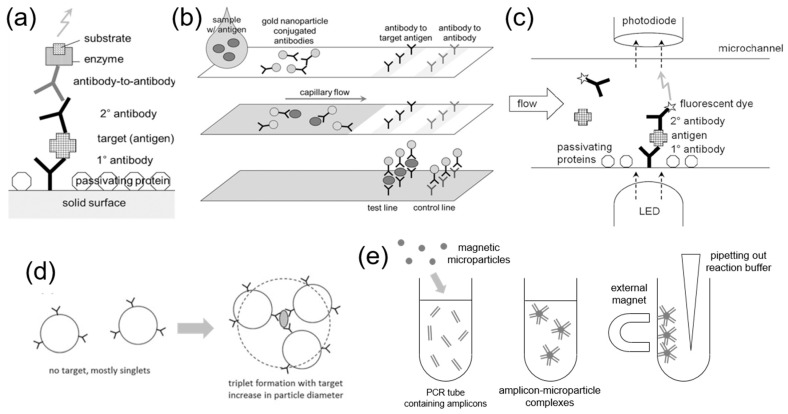
**Working principles of microparticle-based virus detection methods.** (**a**) An ELISA schematic, where the target is sandwiched between primary (1°) and secondary (2°) antibodies. (**b**) Schematic of a lateral flow immunochromatographic assay (LFIA). As capillary flow drives the reaction, the target is sandwiched between two antibodies in the test line, where the pink coloration from AuNPs can be observed. The control line always shows pink if the sample and AuNP-conjugated antibodies flow through the strips. (**c**) A microchannel optical immunosensor schematic. The target is sandwiched between two antibodies, where the secondary antibody is fluorescently labeled. An LED and a photodiode detect such fluorescence. (**d**) Immunoagglutination of antibody-conjugated microparticles in the presence of a target analyte. (**e**) Capturing SARS-CoV-2 amplicons by magnetic microparticles, followed by visual identification. (**a**–**d**) Reprinted with permission from [38]. Copyright 2016, Springer.

Enzyme-linked immunosorbent assay (ELISA) is one of the oldest immunoassay methods, but it is still popular [37]. For detecting virus antigens, primary antibodies are pre-immobilized on the surface of a microwell plate (known as an ELISA plate). A blood serum sample that may contain the virus antigen is added and rinsed from the well. A solution containing secondary antibodies (essentially the same as the primary antibodies) conjugated with the enzyme–substrate pair is added and rinsed. If the virus antigen is present, the entire complex remains in the well, and a microplate reader can quantify the coloration from the enzyme–substrate pair (Figure 2a) [38]. If the virus antigen is absent, the secondary antibody–enzyme–substrate complex is rinsed, and the coloration cannot be observed. Since the target antigen is sandwiched between two antibodies, it is often called a sandwich immunoassay. Lateral flow immunochromatographic assay (LFIA), popularly known as rapid antigen test (RAT), works in a similar principle, where the enzyme–substrate pair is replaced with AuNPs that can exhibit solid pink coloration (Figure 2b) [38]. It is also a type of sandwich immunoassay. Rinsing is achieved automatically via the capillary-action-driven flow through the paper fibers. The LFIA principle has extensively been demonstrated in numerous silicone-based and paper-based microfluidic chips, where other labels, such as QDs, fluorescent dyes, etc., have also been evaluated (Figure 2c) [38]. Other bioreceptors have also been used to detect viruses, like aptamers, enzymes, receptor proteins, etc.

Microparticles are recently gaining popularity in numerous immunoassay platforms, including microplates, LFIAs, and microfluidic chips. They can replace the role of enzyme–substrate pairs, AuNPs, or fluorescent dyes. The substantial size of microparticles may allow them to generate optical signals strong enough for the naked eye to recognize. In addition, antibody-conjugated microparticles can be aggregated together via antibody–antigen binding, known as a particle immunoagglutination assay (Figure 2d). In this platform, immobilizing primary antibodies to a solid surface is unnecessary, e.g., microwell, paper strip, or microfluidic channel, making the reaction occur in one step and eliminating the need for rinsing. Detection can be made using turbidity, nephelometry, light scattering, and microscopic imaging [39,40]. Microparticles can be tagged with fluorescent dyes, and subsequent fluorescence microscopic imaging enables the counting of individual microparticles individually, greatly enhancing the LOD and assay sensitivity [40,41]. Microparticles are also used in other assay platforms that utilize aptamers, enzymes, and protein receptors. 

Microparticles can also be modified with magnetic properties. Ferromagnetic and paramagnetic microparticles exhibit great features for sample separation, sample enrichment, and signal detection. Due to their large surface area, magnetic microparticles can easily capture proteins of interest or amplified products (*amplicons*) from a sample and be physically separated under an external magnetic field (Figure 2e) [42]. Many of these particles’ intrinsic magnetic properties allow their use in signal readouts when paired with magnetic spin detectors, surface plasmon resonance biosensors, and opto-magnetic biosensors [42].

The use of microparticles for virus detection can be classified into four categories:

(1) Label: microparticles are used as labels for virus detection;
(2) Capture: microparticles are used for separating, purifying, capturing, or extracting virus antigens and amplicons from samples;
(3) Both: label and capture;
(4) Other: microparticles are used for other purposes.

For all applications, microparticles must be conjugated with receptors. Based on the receptor type, they can be classified into five categories:

(1) Antibody (to detect viral antigens);
(2) Antigen (to detect neutralizing antibodies);
(3) Aptamer (to detect viral antigens);
(4) Nucleic acid (mainly to capture amplicons);
(5) Other.

In the following sections, we will summarize the use of (polymeric) microparticles for each virus family based on their use (label, capture, both, or other) and the receptor type (antibody, antigen, aptamer, nucleic acid, and other).

## 4. Viruses Detected Using Microparticles

While any virus can be detected using microparticles on various detection platforms, we summarized a list of viruses that have been detected using microparticles. Viruses are infectious microbes ranging in size from 0.02 to 0.3 µm, composed of genomic DNA or RNA and a nucleic protein capsid [43]. These parasites depend on the replication machinery of existing host cells for successful viral infection and replication. The most standard classification of viruses depends on the type of genetic material, between RNA and DNA, and double- and single-stranded. Single-stranded RNA is further categorized into positive- versus negative-sense [44]. 

The five primary transmission routes for viruses include direct contact, aerosols, fomites, ingestion, and vectors [45]. Given the rate and ease of virus exchange between individuals, access to a reliable and accurate viral biosensor is imperative. Without proper preventative control measures or effective diagnostics, viral microbes can lead to wide-scale epidemics and pandemics. Even within the last two decades, the world has witnessed several devastating global health crises led by viral infection outbreaks, including the most recent SARS-CoV-2 pandemic in 2020, the Zika epidemic in 2016, the West African Ebola epidemic in 2014, and the H1N1 pandemic in 2009. Within the scope of this review, the focus will be on biosensors targeting virus families: *Coronaviridae, Filoviridae, Flaviviridae, Hepadnaviridae, Herpesviridae, Orthomyxoviridae, Papillomaviridae, and Retroviridae*.

We searched journal articles for microparticle-based virus detection from the beginning of 2018 to the end of June 2023, i.e., 5.5 years, to focus on the recent trends. The first 2 years represented the pre-pandemic era, while the remaining 3.5 years fell into the pandemic and post-pandemic periods. The target viruses were classified into eight families, as shown below. We could find almost no articles on two virus families—filoviruses and hepadnaviruses—and we had to search further back, to 2015, for these two.

### 4.1. Coronaviridae (Including SARS-CoV-2)

*Coronaviridae* (coronaviruses) is a family of relatively large, enveloped, positive-sense RNA viruses [46], which include the severe acute respiratory syndrome coronavirus (SARS-CoV) and the Middle East respiratory syndrome coronavirus (MERS-CoV). These viruses cause general flu-like symptoms of fever, dry cough, chills, and fatigue, but may also manifest into severe lower respiratory tract infections, such as pneumonia or bronchitis. During viral replication, all members witness the property of RNA polymerase complex jumping during mRNA transcription, which may account for the high mutability of these viruses. 

SARS-CoV-2 is another subtype of coronavirus (resulting in the COVID-19 pandemic), with a 79% sequence similarity with SARS-CoV [47]. Its transmission paths primarily consist of airborne droplets. The spike proteins found on the exterior are responsible for the virus’ invasion of a host cell and are divided into the S1 and S2 regions. The S1 subunit targets the angiotensin-converting enzyme 2 (ACE2) receptor, while S2 facilitates membrane fusion. SARS-CoV-2 patients may be asymptomatic or experience symptoms from acute respiratory distress to life-threatening respiratory failure [47]. Four major vaccines against SARS-CoV-2 currently exist, including Pfizer-BioNTech mRNA, Moderna mRNA, Astra-Zeneca, and Janssen [48]. 

The COVID-19 pandemic prompted a widespread investigation into strategies and technologies to detect and contain SARS-CoV-2. Microparticle-based biosensors have been extensively researched and conjugated with antibodies (to detect viruses) or antigens (to detect neutralizing antibodies). Microparticle-based SARS-CoV-2 detection accounted for the most articles found in our literature survey. These findings may be associated with the frequent rate at which new SARS-CoV-2 variants emerged. Specifically, many purchased antibodies and synthesized antigens could conveniently be used for these new variants, as they are compatible with them; inferior specificity could sometimes become an advantage for a rapidly mutating virus.

Fluorescent polystyrene (PS) microparticles have been prominently utilized for SARS-CoV-2. For example, anti-SARS-CoV-2 IgG and IgM were quantified from serum on fluorescent PS microparticle-based lateral flow immunochromatographic assays (LFIAs) [49]. Variable fluorescence intensities of control and test lines were recorded to confirm the presence of antibodies and quantify them. Similarly, Zhang et al. designed a sensitive sandwich LFIA utilizing fluorescent latex microspheres conjugated with anti-SARS-CoV-2 N protein [50]. In both works, fluorescent signals could be observed with the naked eye or a dedicated sensor with proper excitation (e.g., UV light). The application of fluorescent microparticles grants clear benefits over conventional colorimetric LFIA designs, which often struggle to quantify viral presence. 

**Figure 3:**
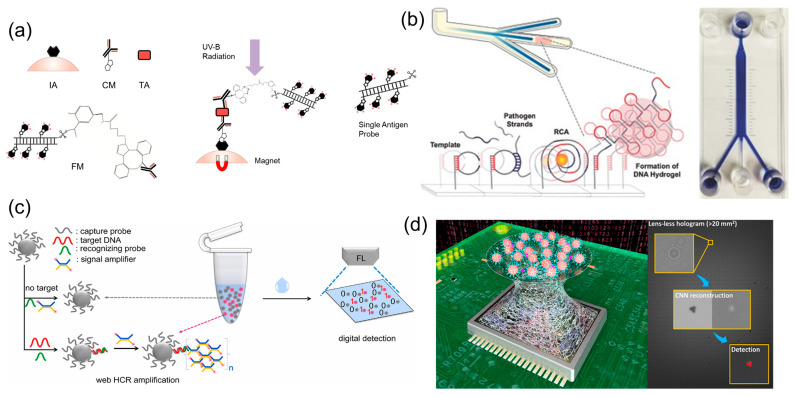
***Label* examples of microparticle-based virus detection.** (**a**) Microparticle-based sensitive fluorescence detection of SARS-CoV-2. Magnetic microparticles are utilized as a solid surface for a sandwich immunoassay, allowing for magnetic separation from fluorescent signals. Reprinted from [51] under Creative Commons Attribution License 4.0 (CC-BY). (**b**) Microparticle-based detection of Ebola virus coupled with rolling circle amplification (RCA). Agglutination of RCA products disrupts the flow of silica microparticles in microfluidic channels. Reprinted with permission from [62]. Copyright 2015, John Wiley & Sons. (**c**) Fluorescent magnetic microparticle array for detecting hepatitis B virus. Reprinted with permission from [82]. DNA probes were coupled to the microparticle surface via EDC chemistry, facilitating the hybridization chain reaction (HCR) of fluorescent probes. Copyright 2023, Elsevier. (**d**) Deep-learning-based lens-free imaging quantifies microparticle aggregation by the human simplex virus. Antibody-functionalized microparticles are clustered around the target antigen, forming imageable particle clusters. Reprinted with permission from [91]. Copyright 2019, American Chemical Society.

Microparticle-based fluorescence detection platforms have another advantage in producing extremely sensitive results. An antibody sandwich design used magnetic microparticles conjugated to a single receptor molecule and achieved single-antigen sensitivity for the multiplexed detection of SARS-CoV-2 and influenza A virus (IAV) (Figure 3a) [51]. Isolation of the fluorescent signals in solution was facilitated by magnetic separation of the photocleaved microparticles. Additionally, studies propose rapid and low-cost serosurveillance strategies that match or surpass the sensitivity of gold-standard methods (i.e., PCR or RT-PCR). A flow-cytometric multiplex microsphere immunoassay detected anti-RBD and anti-N protein in dried blood spots by capturing target antibodies on fluorescent carboxyl beads [52]. Upon comparison to ELISA, the developed assay was more sensitive, while also being more rapid. Fong et al. obtained similar results using a microsphere immunoassay to detect antibodies in clinical serum samples [53].

Magnetic microparticles can also facilitate nucleic-acid-based detection strategies for SARS-CoV-2. For example, amplified products (amplicons) of SARS-CoV-2 were captured on magnetic microparticle surfaces, inducing magnetic-field-induced agglutination of amplicon-captured microparticles. This agglutination makes a signal visible to the naked eye in as little as 20 min [54]. Achieving rapid characterization of amplicon presence is a common obstacle to amplification-based methods. These findings illustrate the possibility of making amplicon detection faster and simpler using magnetic microparticles. Magnetic microparticles can also be used for detecting viral nucleic acids without amplification. Seong et al. developed a method to directly detect the presence of target genes without amplification [55]. By adding silver nanostructures to a magnetic-microparticle-based assay, they analyzed SERS of magnetically isolated target–microparticle complexes and demonstrated impressive sensitivity of SARS-CoV-2 RdRp, E, and N genes. The capture of target genes on microparticle surfaces could also prevent nonspecific binding [56]. Finally, aptamers were also used to detect SARS-CoV-2 antigens instead of antibodies (i.e., amplification-free), where aptamers were conjugated to RNA-driven DNA microparticle motors [57]. Sandwich complexes were formed between aptamer-conjugated microparticle motors and target antigens. Such complex formation impeded the motors’ autonomous movement, enabling detection by smartphone camera motion tracking. 

### 4.2. Filoviridae (Including Ebola Virus)

*Filoviridae* (filoviruses) contains non-segmented negative-sense RNA [58]. The two primary virus types within this family are the Ebola virus and the Marburg virus. Filoviruses are often responsible for fatal hemorrhagic fevers and pathology of the mucous membranes and visceral organs [58]. 

Ebola virus (EBOV) is the most-studied virus among filoviruses. Clinical research has implied that bats may be a potential virus reservoir for EBOV, although it has not yet been established. Viral infection targets mucous membranes, hepatocytes, and renal tubular cells. These account for increased vascular permeability, fluid extravasation, and inhibition of platelet function, which contribute to hypovolemia. Increasing fluid loss can ultimately result in tissue hypoperfusion and multiorgan failure [59]. 

Several microparticle-based Filoviridae biosensors could be found in the literature, where microparticles were primarily used for virus capture. Most methods utilized magnetic microparticles for virus capture and separation, where they were not directly used for detection. 

Two similar biosensors featured microparticle-based sample preparation and antiresonant reflecting optical waveguide (ARROW) quantization. Cai et al. demonstrated the optofluidic device for EBOV detection [60]. Oligonucleotide-labeled magnetic microparticles were used for antigen capture and isolation. Once EBOV DNA annealed to the microparticles and hybridized/unhybridized microparticles were magnetically separated, a thermal shock was provided to unbind captured DNA. This sequestered DNA target was then labeled using SYBR stain. The antigen quantization utilized antiresonant reflecting optical waveguide (ARROW) principles in a microfluidic device. A solid core orthogonally intersected the labeled DNA embedded in a liquid core. When light excitation was introduced, planar refraction allowed the detection of single biomolecules while in flow. Stambaugh et al. also used magnetic microparticles and ARROW [61]. RNA targets of five hemorrhagic fever viruses, Ebola virus (EBOV), Lake Victoria Marburg virus-Angola (MARV), Marburg virus Ravn (RAVN), and Crimean–Congo hemorrhagic fever (CCHF) were differentiated using unique combinations of fluorescent dyes on probe-conjugated capture microparticles. This feature constituted the most significant discrepancy between the two ARROW detectors, and most other methods used were nearly identical, down to the use of a microfluidic chip for ARROW detection. Ultimately, however, the ARROW method was the primary novelty for these articles, while magnetic microparticles were used for standard virus capture and separation. 

Silica microparticles were also used to detect EBOV by the naked eye in conjunction with rolling circle amplification (RCA). Lee et al. demonstrated a lens-free, naked-eye detection of EBOV from RCA in microfluidic channels (Figure 3b) [62]. An RCA template was selectively designed to self-assemble and cross-link with other neighboring templates upon successful binding of target DNA. When placed on a microfluidic platform, a thicker and thicker layer of hydrogel was built up as the RCA cycle repeated until the microfluidic channel was blocked. For visualization of blockage, either bromothymol blue solution or a silica microparticle suspension was added to the channel. Silica microparticles proved advantageous as they were less likely to penetrate the hydrogel barrier. This biosensor shows clear promise for accessible, point-of-care EBOV detection with an approachable quantification method.

Magnetic microparticles were also used to separate or capture EBOV targets, followed by other detection methods. Xu et al. demonstrated a simple EBOV DNA immunoassay with electro-thermal atomic absorption spectrometry [63]. A simple AuNP-based sandwich immunoassay was performed on magnetic microparticles. Following magnetic separation of the unhybridized and hybridized microparticles, aqua regia was applied to dissociate gold ions from the nanoparticles. From there, the electro-thermal atomic absorption spectrometry was measured to detect varying concentrations of gold ions. Sebba et al. demonstrated an immunoassay utilizing unique surface-enhanced Raman scattering (SERS) spectra toward multiplexed detection of EBOV, Lassa fever virus (LASV), and malaria [64]. Three different SERS-active Raman reporters and respective virus antibodies were conjugated to gold nanoparticles to create *nanotags*. Antibody-conjugated magnetic microparticles were used to capture antigens. After mixing these reagents with a whole blood sample for only 25 min, an external magnet helped pelletize the antigens. In detection, only one excitation light could uniquely excite all three nanotags without interfering with the natural absorbance wavelength of whole blood, effectively eliminating the need for sample preparation. 

Another multiplex assay of filoviruses was demonstrated for 10 insect-borne pathogens, including bluetongue virus (BTV), epizootic hemorrhagic disease virus of deer (EHDV), Q-fever pathogen *Coxiella burnetii*, African swine fever virus (ASFV), West Nile fever virus (WNV), Lyme disease pathogen *Borrelia burgdorferi*, vesicular stomatitis virus (VSV), Rift Valley fever virus (RVFV), Ebola virus (EBOV) and Schmallenberg virus (SBV) [65]. Wang et al. developed a relatively simple detection of PCR products using nucleic probes conjugated to fluorescent carboxylated microspheres. Despite the method being standard, 3000 clinical samples were tested to ensure the reliability of results.

### 4.3. Flaviviridae (Including Zika Virus)

The flaviviridae (flavivirus) family consists of arthropod-borne viruses, including dengue virus (DENV), yellow fever virus (YFV), West Nile virus (WNV), Japanese encephalitis virus (JEV), and Zika virus (ZIKV) [66]. Since flaviviruses spread primarily via vector-based transmission, epidemiologists have focused on the effects of increasing population density, rising temperatures, and mass transportation on disease spread. Flaviviruses have positive-sense, single-stranded RNA and cause neurological and hemorrhagic diseases.

Zika virus (ZIKV) belongs to the flavivirus group and is primarily spread through mosquito vectors, breastfeeding, or sexual transmission [67]. Flaviviruses are characterized by positive-sense single-stranded RNA, which releases into the host cell’s cytoplasm after it has been endocytosed. Common symptoms include rash, fever, conjunctivitis, and muscle pain. ZIKV targets neural progenitor cells, manifested as microcephaly in babies that acquire ZIKV. In adults, ZIKV has been demonstrated to increase the likelihood of Guillain–Barre syndrome [67].

Another member of the flavivirus family is the Japanese Encephalitis Virus (JEV), which causes severe neurological disease in humans [68]. Similar to ZIKV, this virus is transmitted through carrier mosquitos and child populations are more susceptible. After infection and replication, the JEV migrates toward brain cells, such as neuronal and microglial cells. Once the virus crosses the blood–brain barrier, severe cases present with paradox, paralysis, Parkinsonian rhythm abnormalities, aberrant posture, seizures, and coma [68].

Hepatitis C (HCV) is a blood-borne RNA virus belonging to the flavivirus family, targeting hepatocytes and B lymphocytes [69]. HCV is a “silent” virus in which the time between infection and observation of apparent symptoms can exceed 30 years in some cases [70]. During its acute phase, hepatitis C is often undiagnosed, as symptoms such as jaundice, fever, and nausea are relatively nonspecific. However, by chronic stages, patients have cirrhosis or hepatocellular carcinoma [69].

Among all virus families, the Flaviviridae family showed the highest occurrence of microparticle usage for both label and capture. A common strategy uses fluorescent magnetic microparticles, where fluorescence is used for high-throughput detection (discrimination among multiple flaviviruses) of target antigens or antibodies from clinical serum samples, and magneticity is used for separating or capturing flaviviruses [71,72,73]. 

**Figure 4:**
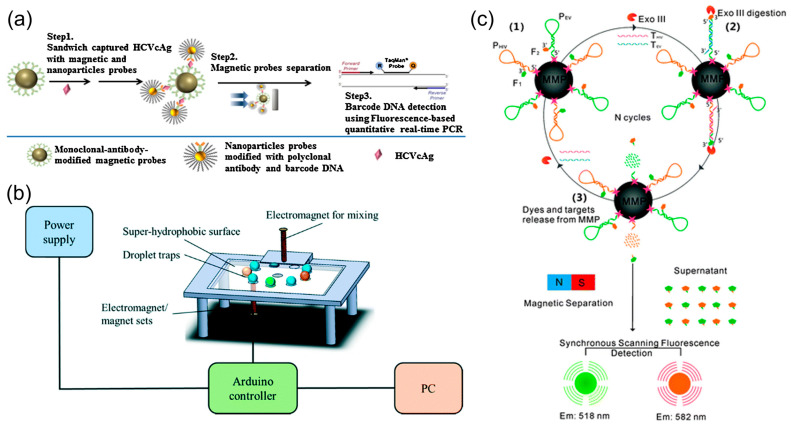
***Capture* examples of magnetic microparticle-based virus detection.** (**a**) Magnetic microparticles are used to capture the hepatitis C virus (HCV) in conjunction with gold nanoparticles (AuNPs). Reprinted with permission from [75]. Copyright 2017, Elsevier. (**b**) Magnetic microparticle-based detection of influenza A on a digital microfluidic platform, where mixing and bead extraction were achieved by an external electromagnet. Reprinted with permission from [97]. Copyright 2020, Royal Society of Chemistry. (**c**) Magnetic microparticle-based detection of human immunodeficiency virus (HIV) and Ebola virus, where the magnetic microparticles conjugated with DNA probes capture the target DNA sequences under external magnetic force. Reprinted with permission from [23]. Copyright 2012, Royal Society of Chemistry.

Furthermore, magnetic microparticles were previously exploited as a pretreatment step for nucleic acid amplification of flaviviruses. Miyachi et al. extracted HCV RNA from serum samples using hybridized magnetic microparticles, allowing target sequences to be isolated from the molecules that could inhibit amplification [74]. Recent work has combined magnetic microparticles with AuNPs to create antibody-sandwich complexes around HCV core antigens (Figure 4a) [75]. Adding AuNP probes allows nucleic acid amplification without isolating genetic material from target antigens. In this application, the advantage of AuNPs has partially been compromised by the difficulty in separating unbound probes, but using additional magnetic microparticles could circumvent this issue. Another detection platform for HCV core antigens was developed by Neves et al., where the magnetic microparticles were combined with flow cytometry technology, illustrating the diverse roles magnetic microparticles may play in detection, even with identical viral targets [76].

Strategies that employ non-magnetic microparticles for flavivirus detection are equally variable. Following successfully capturing JEV antigen on PS microparticles, Liu et al. used acousto-fluidics to isolate antigen–microparticle complexes in a PDMS channel [77]. An oscillatory flow was applied to a particle-trapping microarray, allowing for enhanced enrichment and detection of the anti-Zika NS1 protein via capture on functionalized PS microparticles [78]. It is also possible for polymerized QDs to be conjugated to the Zika NS1 protein for the ZIKV antibody detection, as demonstrated by Rong et al. They successfully detected and quantified ZIKV antibodies from clinical samples using a sensitive and rapid smartphone-based lateral flow assay [79].

### 4.4. Hepadnaviridae (Including Hepatitis B Virus)

Hepadnaviridae is a family of viruses that contain DNA nucleic information replicated by reverse-transcriptases for the infection of hepatocytes [80]. Viruses belonging to the Hepadnaviridae family include the hepatitis B virus (HBV), although not the hepatitis C virus (HCV; belongs to the Flaviviridae family), despite its naming convention. 

Hepatitis B (HBV) is a covalently closed circular DNA virus that evades the innate immune response, delaying symptom presentation. Similar to hepatitis C, HBV is spread through blood-borne pathogens, although hepatitis B has demonstrated a higher transmission rate than hepatitis C [81]. This liver inflammatory disease in chronic conditions can result in cirrhosis or hepatocellular carcinoma.

Various aspects of microparticles have been utilized for HBV biosensing. For instance, the high surface area of carboxyl-functionalized magnetic beads was used to densely hybridize HBV DNA probes for an isothermal web hybridization chain reaction (Figure 3c) [82]. Strong fluorescent signals could be obtained from the amplified complexes on microparticles, achieving a detection limit of 5 fM HBV. Moreover, this method allowed the reactions to be conducted in microliter-scale volumes. Xu et al. investigated the clinical applications of this microparticle-enhanced hybridization chain reaction, achieving a sensitivity of five HBV copies per reaction from serum samples [83]. Non-magnetic microparticles were also used for fluorescence-based detection of HBV DNA [84]. In this study, the microparticle-based DNA walker generated negligible background fluorescence, permitting the precise isolation of target signals. 

Polymerized QDs were also used for multiplexed detection of HBV. When used in tandem, the potent fluorescence of QDs and the target agnostic surface functionalization of polymeric microparticles could generate an individualized signal for single targets. In this manner, multiple targets could be detected from one solution. Using this method, Kim et al. attained a clinical sensitivity range of 80.4–90.5% for multiplexed HBV genome detection [85]. The other two groups also distinguished between several HBV surface antigens and antibodies, utilizing various polymerized QDs [86,87].

### 4.5. Herpesviridae (Including Herpes Simplex Virus)

The herpesviridae (herpes virus) family are enveloped, double-stranded DNA pathogens with extracellular glycoproteins for host transduction [88]. Herpesviruses have lytic and latent phases and are highly adapted to their hosts. Severe pathology is observed only in the youth, elderly, and immunocompromised [89]. 

Herpes simplex virus (HSV) is a double-stranded DNA virus categorized further into HSV-1 and HSV-2. HSV infection is lifelong, with outcomes ranging from asymptomatic to life-threatening [90]. Following the primary invasion of mucosal epithelial cells, HSV establishes latency in the neurons of the peripheral nervous system. From there, the virus may remain latent, or specific triggers may cause reactivation of the virus [90]. Regardless, HSV infection is a significant risk factor for developing Alzheimer’s disease and other neurodegenerative diseases. 

Among the microparticle-based biosensors in our literature survey, the herpesviruses accounted for the smallest number. Furthermore, only one of the three articles featured singleplex herpes virus detection. This suggests the need for further investigation into microparticle biosensing of the herpes virus. 

Wu et al. proposed a deep-learning analysis of microparticle aggregation induced by HSV (Figure 3d) [91]. Microparticles were coated with anti-HSV, creating aggregated clusters when exposed to the target HSV. Instead of conventional microscopic observation, they captured lens-free holographic images and reconstructed their high-resolution images via 3D deep learning. Detection limits reached 4.91 viral copies/μL, or 25 viruses/test.

In contrast, a multiplexed assay developed by Tao et al. featured HSV detection among five other virus targets [92]. This biosensor’s distinct character was co-localization, the overlap of two uniquely fluorescent microparticles to produce a new distinguishing label. Four distinctly colored microparticles were each conjugated to three different antigen-complementary nucleic sequences, making 12 probe combinations. Six different viruses were detected from the unique combination of two colored microparticles. Although fluorescence-based microparticle labeling has commonly been used, this co-localization assay could effectively tailor multiplex detection. 

In yet another multiplex biosensor, 14 respiratory virus plasmids, including HSV and Epstein–Barr virus (EBV), were simultaneously detected by Shi et al. [93]. Following sample amplification using PCR, viral sequences were detected using colored PS microparticles conjugated to complementary nucleic acid primers called *anti-TAGs*. Limits of detection across the 14 plasmids ranged from 10^3^ copies/reaction to 50 copies/reaction. Given the assay’s target-agnostic nature and relative specificity, this is ideal for high-throughput screening. Compared to the other multiplexed biosensors of our review, Shi et al.’s method boasted the highest number of different targets that span multiple virus families. Such highly specific multiplex platforms hold considerable potential for the next generation of microparticle-based virus detection. 

### 4.6. Orthomoxyviridae (Including H1N1 Swine Flu Virus)

Orthomyxoviridae is a family of RNA-based viruses, including seven genera [94]. Four of these are responsible for influenza disease in humans. In particular, influenza A subtype H1N1 caused the majority of historical pandemics, including the swine flu H1N1 outbreak in 2009 and the Spanish flu H1N1 pandemic in 1918. Influenza A and B viruses contain segmented negative-sense RNA with either hemagglutinin (HA) or neuraminidase (NA) surface antigens. An infected patient suffers damage to the respiratory epithelium and bronchi, which may result in eventual respiratory failure [95]. 

H1N1 swine flu is a subtype of the influenza A virus and causes infection of the host’s upper, and potentially lower, respiratory tracts [96]. While the virus originated in pigs (thus the name *swine*), it has since undergone recombination to become a human threat, especially with the emergence of the novel H1N1 flu in 2009. The virus is most likely transmitted through airborne droplets, but can be shared through direct contact. While most symptoms are similar to those of other influenza, a severe case of H1N1 may result in respiratory failure and subsequent death [96]. The US Food and Drug Administration (FDA) has approved a vaccine specific to H1N1 swine flu since 2009. 

Numerous microparticle-based biosensors have been developed for various influenza A viruses, while our survey indicated a relatively even distribution between label, capture, and other functions. Nearly half of the assay emphasized magnetic or thermal properties to create simple and rapid detection with minimal user intervention [97,98].

Two articles showed the magnetic features of microparticles. A microfluidic platform proposed by Lu et al. used electromagnetic forces on magnetic microparticles to automate an ELISA-based H1N1 immunoassay [97]. Droplets with magnetic microparticles are manipulated on a highly hydrophobic surface, exposing samples to sequential ELISA reagents and washing steps (Figure 4b). This condensed version of microparticle immunoassay cut assay time down to 40 min from sample load to detection. Garbarino et al. integrated rolling circle amplification (RCA) into a microfluidic lab-on-a-chip platform for influenza B viruses [98]. The device housed three sequential chambers loaded with reagents for target annealing, rolling circle amplification, and optomagnetic detection. Target DNA complexes were captured by probe-conjugated magnetic microbeads, which are essential in driving the progression of this assay through each reaction chamber. 

Other articles showed assay quantification. Zhang et al. developed a unique photonic (PhC) barcode multiplex immunoassay targeting H1N1, H5N1, and SARS-CoV-2 [99]. PhC barcodes comprised colored microspheres decorated by self-polymerizing polydopamine and fluorescein isothiocyanate-conjugated (FITC) antibodies. Photoluminescence was quenched with the target presence, where FITC antibodies dissociated and bound to the virus antigen. Although fluorescence-based detection methods have gained considerable favorability, colorimetric detection offers simple visualization without the need for an excitation light. A colorimetric sandwich complex assay for H3N2 viruses has been developed by Chen et al. [100]. H3N2-specific aptamers were conjugated to magnetic microparticles for antigen capture. Subsequently, glucose-oxidase AuNPs were bound to target antigens using concanavalin A, a selective glycan-binding protein. After exposure of this sample to glucose, reduction–oxidation reactions generated either red- or blue-presenting nanoparticles, corresponding to the absence or presence of the target, respectively [101]. This binary assay offers rapid and straightforward virus detection at the expense of finite quantification. Krejcova et al. used electrochemical detection to identify the H1N1, H3N2, and H5N1 influenza subtypes [102]. They used probe-conjugated paramagnetic microparticles to capture and isolate three influenza oligonucleotides. Then, metallic QDs conjugated with anti-sense DNA were added for hybridization. Square wave voltammetry could detect the antigen presence utilizing the electrochemical properties of QDs. 

Some detection methods significantly utilized the cross-molecular reactions between captured virus targets. Matsubara et al. used a hemagglutination inhibition assay on a glycan-functionalized microparticle to detect influenza virus [101]. The principle depended on the binding between the virus hemagglutinin and sialic acid glycan receptors on red blood cells. Red blood cells were substituted with sialyl- and sulfated-lactose glycan moiety-conjugated microparticles in this biosensor. 

### 4.7. Papillomaviridae (Including Human Papillomavirus)

Small, non-enveloped, double-stranded DNA viruses comprise the Papillomaviridae family (papillomaviruses) [103]. They frequently target epithelial cells and successfully evade the activation of the innate immune system. This family’s only known human-infecting virus member is human papillomavirus. 

Human papillomavirus (HPV) is a double-stranded DNA virus causing proliferative lesions of the cutaneous and mucosal epithelium [104]. HPV infection is particularly focused as it has been identified as a significant precursor to cervical carcinoma. Integration into the epithelial cell differentiation cycle allows for the virus’ massive proliferation. HPV is spread through sexual transmission and can be prevented through a preventative HPV vaccine. 

The number of journal articles about the microparticle-based detection of papillomaviruses is relatively low compared to other virus families cataloged in this review. This low number may warrant more thorough investigations of microparticle-based strategies. However, even among a limited pool of detection platforms, microparticles displayed various functions. 

Ultrasensitive and multiplexed detection of HPV DNA was achieved using PS microparticles synthesized in-house, modified with a silica coating, and doped with an organic dye [105]. Organic dyes produce intense fluorescent signals even in minute concentrations, but sensitivity to the solution environment can be an inhibiting factor [106]. Encasing organic-dye-doped microparticles in silica shells could potentially stabilize dye behavior in solution. Although commercial vendors are convenient channels for acquiring pre-synthesized microparticles, manual microparticle synthesis provides blank canvases for tailored detection platforms. On the other hand, commercial fluorescent microparticles can offer affordable biosensors, and their diverse selection of fluorescent dyes makes it possible to retrofit microparticles into a sensor’s design. For example, Obahiagbon et al. created a low-cost and portable detection platform for the HPV antigen by measuring the fluorescent intensity of latex microspheres encapsulating Nile red dye, which were chosen for their compatibility with the excitation spectrum of LEDs [107].

Magnetic PS microparticles can play diverse roles in non-amplification-based HPV nucleic acid detection, credited to their ability to immobilize and manipulate captured targets. Bartosik et al. captured HPV-16 and HPV-18 DNA on a magnetic PS microparticle [108]. Target–microparticle complexes were immobilized onto a bare electrode, which was detected electrochemically. Xiang et al. employed the magnetic mixing abilities of DNA probes on PS microparticles to facilitate binding events with hybridized QDs, allowing fluorescent detection of HPV-16 and HPV-18 [109].

### 4.8. Retroviridae (Including Human Immunodeficiency Virus)

Viruses in the Retroviridae family (retroviruses) are characterized by their single-stranded RNA nucleic information dependent on DNA polymerase for their *retroviral* replication [110]. Further classification can be made into two subfamilies, Orthoretrovirinae and Spumaretrovirinae, and seven genera [111]. cDNA translated from viral positive-sens RNA is integrated into host genomes and manifests into immunological diseases such as lymphomas, leukemias, and neuro deficiencies [112]. 

Human immunodeficiency virus (HIV) is an infection in which the victim’s immune system is targeted, especially CD4 lymphocytes, monocytes, and thymocytes. In the early stages of HIV infection, also known as acquired immune deficiency syndrome (AIDS), patients experience influenza-like symptoms. However, as leukocyte counts continue to drop, the patient becomes severely vulnerable to life-threatening infections [111,112]. 

Microparticle-based biosensors for retroviruses have demonstrated label, capture, and their combination functions. Yuan et al. developed a nucleic acid detection circuit called a *DNA walker* that features a spontaneous intramolecular hybridization reaction and subsequent carboxyfluorescein cleavage by endonucleases [24]. A fluorescent single-strand HIV oligonucleotide and a nucleic acid probe were conjugated to a microparticle. Upon successful target HIV DNA binding, a cascade of entropy-driven reactions activated nicking endonuclease (Nb.BbvCI), recognizing the specific base sequences in the hybridized sandwich complex. Cleavage at these points released carboxyfluorescein mononucleotide signal reporters. This target binding and fluorescence release cycle was repeated, allowing for a large, quantifiable amount of signal reporters in each sample. This highly specialized biosensor could distinguish negative samples down to a one-base mismatch. 

A similar biosensor demonstrated by Luo et al. capitalized on fluorescent oligonucleotide cleavage using nonspecific exonuclease III instead [23]. Two distinctly colored fluorogenic DNA probes targeting HIV and EBOV antigens were conjugated to magnetic microparticles. When either target DNA hybridized, non-sequence-specific exonuclease III cleaved off a mononucleotide at the exposed 3′ terminus, releasing a fluorophore (Figure 4c). Like the previously introduced assay, this process cyclized as the target antigen continued to reanneal to microsphere probes, and fluorophores were cleaved off. Lastly, an external magnetic field was applied to remove the magnetic microparticles for sample isolation and analysis. 

The following three assays utilize ELISA-like immunoassay principles. Wang et al. proposed a relatively simple lateral flow immunochromatographic assay (LFIA) targeting the p24 protein, forming the protective shell surrounding the HIV core [113]. The detection microparticle was fluorescently labeled. Sher et al. captured the HIV-1 antigen on an antibody-coated glass chip, then labeled it using high refractive index microparticles [114]. They used a digital camera (CMOS image sensor) to detect the presence of these high-refraction microparticles, and the results were fed into a calibrated deep-learning algorithm to determine a final viral count. They used holography-based lens-free imaging, similar to the method demonstrated by Wu et al. [91]. Miyagawa developed a sandwich assay of retroviruses, utilizing an ultrasensitive cold atom gravimeter (CAG)-based sensor. Following HIV-2 DNA capture by paramagnetic microparticles, the target was labeled by an AuNP-reporter oligonucleotide sequence. Positive samples were determined based on their relative density measured by CAG [115].

## 5. Classification of Microparticle-Based Virus Detection Methods

Table 1 summarizes various microparticle-based virus detection methods described in the previous section, categorized by their use (label, capture, or other) and the receptor type (antibody, antigen, aptamer, nucleic acid, and other). The size of microparticles and the limits of detection (LODs) are also summarized.

**Figure 5:**
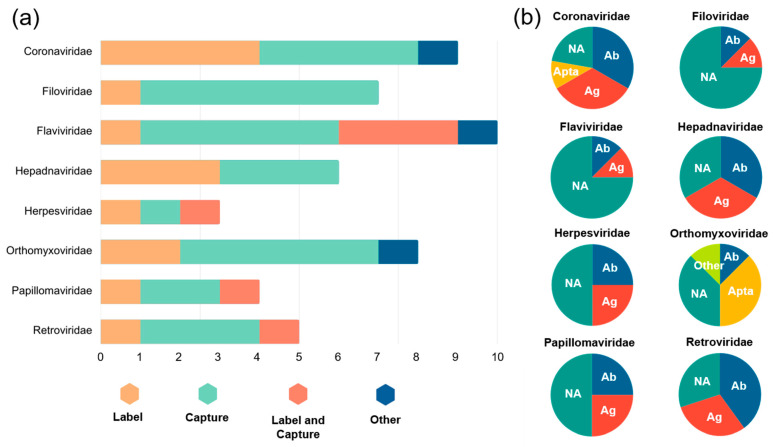
**Graphical classifications of microparticle-based virus detection methods.** (**a**) Categorized by the use of microparticles for eight virus families. X-axis represents the number of journal particles. (**b**) Categorized by the receptor types on microparticles for eight virus families. Ab = antibody, Ag = antigen, Apta = aptamer, NA = nucleic acid.

The number of articles is plotted against eight different virus families in Figure 5a. Coronaviridae, Flaviviridae, and Orthomyxoviridae make up the greatest numbers, representing the SARS-CoV-2 pandemic, Zika virus pandemic, and H1N1 swine flu pandemic, respectively, that have affected us in the last decade. Microparticles were predominantly used to capture Flaviviridae (including Zika virus) and Orthomyxoviridae (including H1N1). However, with the recent COVID-19 pandemic, microparticles were equally used for label and capture for detecting SARS-CoV-2. Interestingly, Flaviviridae is the only major family where microparticles were utilized for both label and capture.

The types of receptors are also shown as pie charts in Figure 5b, sorted by virus families. Coronaviridae (including SARS-CoV-2) utilized primarily antibodies and antigens, where viral antigen assay and neutralizing antibody assay are equally important. Similar trends can be seen with Hepadnaviridae (including hepatitis B virus), Herpesviridae (including herpes simplex virus), Papillomaviridae (including human papillomavirus), and Retroviridae (including HIV). Nucleic acids (primarily via amplification) were used throughout all virus families, particularly most prevalent among Filoviridae (including Ebola virus) and Flaviviridae (including Zika virus). Aptamers were not used often, only for the Orthomyxoviridae family (including H1N1), potentially suggesting a need for future work.

## 6. Concluding Remarks and Future Directions

Our survey results demonstrate the diverse application of microparticles towards various virus detection platforms. Nucleic acid amplification methods, immunoassays, and general hybridization reactions benefit from the high surface area-to-volume ratio of microparticles interacting with virus targets. External manipulation of magnetic microparticles presents unique strategies for mixing, separating, and immobilizing captured analytes. Finally, the convenient size of polymeric microparticles allows centrifugal separation and imaging by conventional microscopes, simplifying reaction or detection procedures. These characteristics make microparticles ideal for rapid, point-of-care diagnostic applications, which are critical for maintaining global health. 

Microparticle-based detection platforms still face limitations regarding their signal transduction capabilities. Within our search, electrochemical detection using microparticles remains largely uninvestigated. Certain virus families, namely Herpesviridae, Papillomaviridae, and Retroviridae, have little research into detection through microparticle-based methods. Moreover, the Filoviridae and Papillomaviridae families are highly dependent on nucleic-acid-based detection, indicating that there may be a gap in research involving antibody- and antigen-based methods for these virus families. Across all virus families, microparticles have been highly exploited for uses in analyte capture, being used in a higher or equal proportion to microparticles as labels. The ability to directly label target analytes has the potential to produce strong signals. However, labeling microparticles depends mainly on fluorometric detection. Further studies into microparticle-based virus biosensors should seek to close these discontinuities in research.

## Figures and Tables

**Table 1:** Summary of various microparticle-based virus detection methods.

Virus	Microparticle Use			Microparticle Character				LOD	Refs.
	Label	Capture	Other	Properties	Material	Receptor	Size		
*Coronaviridae*									
SARS-CoV-2	✓			Fluorescent	Latex	Antibody	NA	100 ng/mL	[50]
	✓				NA	Antigen	NA	NA	[52]
	✓				PS	Antigen	NA	NA	[49]
		✓		Magnetic	NA	Nucleic acid	2.8 µm	1.0 fM	[55]
		✓			PS	Nucleic acid	2.8 µm	NA	[54]
		✓		Nonmagnetic	PS	Antigen	4.95 µm	NA	[53]
SARS-CoV-2, IAV *		✓		Magnetic	PS	Antibody	NA	30 ng/mL	[51]
			✓	Nonmagnetic	DNA	Aptamer	5 µm	10^3^ copies/mL	[57]
*Filoviridae*									
EBOV		✓		Magnetic	NA	Nucleic acid	NA	3.0 pM	[63]
		✓			PS	Nucleic acid	1 µm	0.2 PFU/mL	[60]
		✓		Nonmagnetic	Hydrogel	Nucleic acid	NA	NA	[62]
EBOV, BTV, ASFV, RFV, SBV, WNV *	✓			Fluorescent	PS	Nucleic acid	NA	10 copies/rxn	[65]
EBOV, MARV, CCHF		✓		Magnetic	NA	Nucleic acid	NA	NA	[61]
EBOV, LASV, Malaria		✓			NA	Antibody	NA	10^5^ PFU/mL	[64]
*Flaviviridae*									
JEV		✓		Nonmagnetic	PS	Antibody	NA	10^3^ PFU/mL	[77]
ZIKV	✓			Fluorescent	Carboxyl polymer	Antibody	1 µm	0.045 ng/mL	[79]
		✓		Nonmagnetic	PS	Antigen	20 µm	1 ng/mL	[78]
ZIKV, DENV1–4, WNV	✓	✓		Fluorescent, magnetic	PS	Antigen	NA	NA	[71]
ZIKV, DENV1–4	✓	✓			PS	Antigen	NA	NA	[72]
ZIKV, YFV	✓	✓			PS	Antibody	6.5 µm	1.88–2.77 fM	[73]
HCV		✓		Magnetic	NA	Nucleic acid	NA	NA	[74]
		✓			PS	Nucleic acid	2.8 µm	1 fg/mL	[75]
		✓			Silica	Nucleic acid	1 µm	NA	[76]
*Hepadnaviridae*									
HBV	✓			Fluorescent	PS ^1^	Antibody, antigen	6.6 µm	NA	[86]
	✓				Carboxyl PS ^2^	Antibody, antigen	NA	NA	[87]
	✓				PS ^3^	Nucleic acid	2.7, 3.5 µm	NA	[85]
		✓		Magnetic	Carboxyl beads	Nucleic acid	2.7 µm	NA	[82]
		✓			NA	Nucleic acid	5 µm	5 copies/rxn	[83]
		✓		Nonmagnetic	NA	Nucleic acid	1 µm	0.20 nM	[84]
*Herpesviridae*									
HSV		✓		Nonmagnetic	Silica	Antibody	2 µm	5 copies/µL	[91]
HAV, HBV, HCV, HIV, HPV, HSV	✓			Fluorescent	PS	Nucleic acid	1 µm	0.6–3 fM	[92]
HSV, VZV, CMV, EBV, MuV, MeV, JCV, JEV *, CA16, EV71, ECHO, HEV	✓	✓		Fluorescent, Magnetic	PS	Nucleic acid	NA	NA	[93]
*Orthomyxoviridae*									
H1N1, H1N2, H3N2	✓			Dyed	PS	Oligo-saccharide	1 µm	200 PFU	[101]
H1N1, H5N1, SARS-CoV-2 *	✓			Fluorescent	Silica ^4^	Antibody	200 µm	0.2 ng/mL	[99]
H3N2		✓		Magnetic	MMP	Aptamer	1.02 µm	11.6 µg/mL	[100]
H1N1, H5N1, H3N2		✓			PS	Nucleic acid	NA	0.5–1.0 ng/mL	[102]
H1N1		✓			PS	Aptamer	1.05 µm	0.032 HA units/rxn	[97]
IBV		✓			PS	Nucleic acid	1 µm	2 pM	[98]
*Papillomaviridae*									
HPV	✓			Fluorescent	Latex	Antibody	1 µm	NA	[107]
	✓	✓		Fluorescent	PS ^5^	Nucleic acid	2.5 µm	1 fmol/mL	[105]
		✓		Magnetic	PS	Nucleic acid	2.8 µm	NA	[108]
		✓		Magnetic	PS	Nucleic acid	1 µm	6 × 10^−11^ M	[109]
*Retroviridae*									
HIV	✓	✓		Fluorescent	NA	Nucleic acid	1 µm	2 pM–5 nM	[24]
HIV-1	✓				NA	Antibody	0.1–100 µm	3.4 pg/mL	[113]
HIV, EBOV *		✓		Magnetic	PS	Nucleic acid	1.02 µm	70 pM	[23]
HIV-1		✓		Nonmagnetic	PS	Antibody	3, 5, 7 µm	1569 virions/mL	[114]
HIV-2		✓			PMMA	Nucleic acid	6.33, 9.57 µm	NA	[115]

Note: An asterisk next to the name of a virus indicates that it was detected using the same platform, while not being a part of the virus family listed above. NA = information not available. All microparticles were acquired from commercial vendors unless otherwise specified. A superscript number next to the microparticle material indicates that the microparticle was synthesized in-house: ^1,2^ porous glass membrane emulsification, ^3^ droplet-based synthesis, ^4^ microfluidic droplet-based synthesis, ^5^ dispersion polymerization and sol–gel process.

## Data Availability

Not applicable.

## Abbreviations

ACE2 = angiotensin-converting enzyme 2; ARROW = anti-resonant-reflecting optical waveguide; ASFV = African swine fever virus; AuNP = gold nanoparticle; BTV = bluetongue virus; CA16 = coxsackie A virus type 16; CAG = cold atom gravimeter; CCHF = Crimean–Congo hemorrhagic fever; CIA = chemiluminescent immunoassay; CMOS = complementary metal oxide semiconductor; CMV = cytomegalovirus; COVID-19 = coronavirus disease 2019; DENV = dengue virus; EBOV = Ebola virus; EBV = Epstein–Barr virus; ECHO = echovirus; EDC = 1-ethyl-3-(3-dimethylaminopropyl)carbodiimide; EHDV = epizootic hemorrhagic disease virus of deer; ELISA = enzyme-linked immunosorbent assay; EV71 = enterovirus type 71; HA = hemagglutinin; HAV = hepatitis A virus; HBV = hepatitis B virus; HCR = hybridization chain reaction; HCV = hepatitis C virus; HEV = human enterovirus; HIA = hemagglutination-inhibition assay; HIV = human immunodeficiency virus; HPV = human papillomavirus; HSV = herpes simplex virus; IAV = influenza A virus; IBV = influenza B virus; IgG = immunoglobulin G; IgM = immunoglobulin M; JCV = JC virus; JEV = Japanese encephalitis virus; LAMP = loop-mediated isothermal amplification; LASV = Lassa fever virus; LFIA = lateral flow immunochromatographic assay; LOD = limit of detection; MARV = Victoria Marburg virus-Angola; MERS-CoV = Middle East respiratory syndrome coronavirus; MeV = measles virus; MuV = mumps virus; NA = neuraminidase; PCR = polymerase chain reaction; PDMS = polydimethylsiloxane; PMMA = polymethylmethacrylate; POC = point of care; PS = polystyrene; QD = quantum dot; RAT = rapid antigen test; RAVN = Marburg virus Ravn; RBD = receptor-binding domain; RCA = rolling circle amplification; RFV = Rift Valley fever virus; RT-LAMP = reverse transcription − loop-mediated isothermal amplification; RT-PCR = reverse transcription − polymerase chain reaction; RVFV = Rift Valley fever virus; rxn = reaction; SARS-CoV-2 = severe acute respiratory syndrome coronavirus 2; SBV = Schmallenberg virus; SERS = surface-enhanced Raman spectroscopy; VSV = vesicular stomatitis virus; VZV = varicella-zoster virus; WNV = West Nile virus; YFV = yellow fever virus; ZIKV = Zika virus.
